# Understanding foreign language writing anxiety and its correlates

**DOI:** 10.3389/fpsyg.2022.1031514

**Published:** 2022-11-03

**Authors:** Rui Li

**Affiliations:** School of Foreign Languages, Hunan University, Changsha, China

**Keywords:** correlate, foreign language, meta-analysis, writing anxiety, self-efficacy

## Abstract

Despite the increasing number of empirical studies that investigated foreign language writing anxiety and its correlates, there is still a lack of quantitative meta-analytic attempt on the effect sizes among these studies. To bridge the gap, this study identified 84 effect sizes from 22 primary studies to meta-analyze the correlations of foreign language writing anxiety and several key high-and low-evidence correlates. For the two high-evidence correlates, moderator analyses were also conducted, which demonstrated that foreign language writing anxiety has a moderate correlation with foreign language writing self-efficacy and foreign language writing performance. The three low-evidence correlates have positively moderate effects of foreign language listening anxiety, foreign language speaking anxiety and foreign language reading anxiety. The significant moderating effects of learners’ age and language proficiency were obtained. With respect to the results, pedagogical implications were discussed as well.

## Introduction

As one of the important productive language skills, writing skill receives considerable attention in second language acquisition (SLA) and evaluation (Rakedzon and Baram-Tsabari, 2017). However, due to second or foreign language writing anxiety/apprehension, language learners may encounter writing difficulties and feel cognitively and physiologically nervous when writing in a foreign language, as reflected from the decreased writing performance and negative writing affects (Abdel Latif, 2015, 2019; He, 2018; Russell-Pinson and Harris, 2019).

Foreign language writing anxiety is often defined as “the dysfunctional anxiety that many individuals suffer when confronted with foreign language writing tasks” (Cheng, 2002, p. 647). In other words, highly anxious learners are documented to achieve lower foreign language performance (Abdel Latif, 2015), poorer foreign language writing performance (Cheng, 2002, 2004), and lower foreign language writing affects, such as motivation (e.g., Alico, 2016; Tsao et al., 2017; Abdel Latif, 2019), writing self-efficacy (e.g., Cheng, 2004; Woodrow, 2011; Abdel Latif, 2019), writing attitude (Sarkhoush, 2013), and writing strategies (Wu and Lin, 2016; Tsiriotakis et al., 2017). Despite these numerous empirical studies, the accumulation of these studies necessitates research on the related factors of foreign language writing anxiety from a more generalizable meta-analytic approach.

## Literature review

### Related studies of foreign language writing anxiety

In the literature, subsequent to preliminary conceptual work of first language (L1) Writing Apprehension Test (WAT) produced by Daly and Miller (1975), an emerging array of second language (L2) studies (Cheng, 1998, 2002, 2004, 2017; Cheng et al., 1999) have begun to offer empirical insights into foreign language writing anxiety. For instance, Cheng (2004) aimed to develop and measure the reliability and validity of the Second Language Writing Anxiety Inventory (SLWAI) among 421 Chinese English-as-a-foreign-language (EFL) learners. Result of explanatory factor analysis supports a three-factor constructs: avoidance behavior, cognitive anxiety and somatic anxiety. More specifically, avoidance behavior represents “an indicative of avoidance behavior,” cognitive anxiety is defined as “anxiety related to fear of negative evaluation or worrisome perceptions,” and somatic anxiety refers to “anxiety related to increased physiological arousal” (Cheng, 2004, p. 325).

Apart from the empirical attempts, qualitative literature reviews on foreign language writing anxiety have also been recently presented (Ma and Dong, 2018; Abdel Latif, 2019). For instance, Ma and Dong (2018) performed a review of foreign language writing anxiety by retrieving all the related studies published in Chinese key journals from 2001 to 2015, and identified two major findings pertinent to the study: First, most of the existing studies focused on exploring the relationships between foreign language writing anxiety and its related correlates, *viz*. foreign language writing performance, affects and other related anxieties. Second, those studies published to date also obtained that the relationships were modulated by some contextual-related and learner-related variables, such as types of anxiety, language distance, target language, learners’ age and foreign language proficiency. In a more recent study, Abdel Latif (2019, p. 8) critically and systematically reviewed key writing motivational constructs from the literature, and highlighted the need to “make the results of a particular study more generalizable.”

### Related meta-analyses

While these empirical attempts and qualitative literature reviews may shed some light on foreign language writing anxiety research, the aggregated effects regarding correlates of foreign language writing anxiety remain largely unidentified. As such, a closer look into the research domain has been made, which reveals no meta-analysis of foreign language writing anxiety published to date, but similar meta-analyses (e.g., Teimouri et al., 2019; Zhang, 2019; Botes et al., 2020; Li, 2022a) pertinent to the study. For instance, a meta-analysis of 97 effect sizes conducted by Teimouri et al. (2019) indicated a moderate and negative correlation (*r* = −0.360) between foreign language anxiety and foreign language performance. Moderating effects regarding types of language performance, educational level, types of anxiety were also achieved. Likewise, Zhang (2019) also obtained the moderate, negative correlation (*r* = −0.340) between foreign language anxiety and foreign language performance. Apart from the moderators mentioned in Teimouri et al. (2019), language distance has also been found to significantly moderate the foreign language anxiety–foreign language performance correlation. Similarly, another meta-analysis reported by Botes et al. (2020) dealt with the correlation of Foreign Language Classroom Anxiety (FLCA) and five types of academic performance, including general language performance and four skill-specific (listening, speaking, reading and writing) performance. Concerning the result, moderately negative correlations have been achieved regarding FLCA and all types of academic performance. To the best of our knowledge, only Li’s team (Li, 2022a,b) began to meta-analyze correlates of foreign language reading and listening anxiety, warranting a fresh look at other skill-specific anxieties, e.g., foreign language writing anxiety. Because it would be of vital importance to gain a deeper understanding of the correlates of foreign language writing anxiety, and earlier studies (Pae, 2013; Chen, 2019) on foreign language writing anxiety also argue that it is distinguishable from the domain-general foreign language anxiety. For instance, Pae (2013) aimed to revisit the relationship between four skill-specific anxieties and the domain-general foreign language anxiety. A multiple regression analysis indicated that foreign language writing anxiety could only explain 9.5% variance of foreign language anxiety (*β* = 0.095, *p* = 0.041, cf. Table 1, Pae, 2013, p. 248), suggesting that both are statistically distinguishable from each other. On the other hand, existing studies on foreign language anxiety focus too much on “test anxiety and general trait anxiety” (Chen, 2019, p. 314), which may fail to assess language learners’ responses to the skill-specific foreign language writing anxiety.

**Table 1:** Overall average correlations and publication bias test for the low-evidence correlates.

Correlates	*k*	*r* [95% CI]	*Q*	*I^2^*	*N_fs_*	*N_observed_*	*r_adjusted_*
Listening anxiety	5	0.485 [0.415, 0.549]	5.289	24.366	251	5	0.481 [0.430, 0.528]
Speaking anxiety	9	0.455 [0.377, 0.526]	21.358**	62.544	685	9	0.469 [0.426, 0.509]
Reading anxiety	5	0.489 [0.376, 0.588]	12.258	67.367	277	5	0.532 [0.482, 0.578]

** *p* < 0.010.

Taken together, although the related meta-analytic studies have been valuable to gain an understanding, yet indirect, of fundamental aspects of foreign language writing anxiety, little is still known about its main correlates (e.g., foreign language writing performance, foreign language writing self-efficacy, foreign language listening anxiety, foreign language speaking anxiety and foreign language reading anxiety), and potential moderators (e.g., types of anxiety, age, target language, language proficiency and language distance), calling for research into the possible correlates and moderators of foreign language writing anxiety.

### Related correlates and moderators

Currently, foreign language writing anxiety studies have focused on identifying its correlates, including foreign language writing performance (Guo and Fan, 2009), foreign language writing self-efficacy (Tola and Sree, 2016; Guo, 2018), foreign language listening anxiety (Xiao and Wong, 2014), foreign language speaking anxiety (Gkonou, 2011) and foreign language reading anxiety (Cheng, 2004), respectively.

Drawing on Li (2022a), the identification of correlates should observe the following steps: First, prior to meta-analysis, an initial search should be conducted to exhaustively identify all the related correlates. Second, those correlates of very low numbers of effect sizes (*k* < 3, see also Li, 2022a) should be removed, as they are insufficient for generating trustworthy interpretations. Normally, to make the meta-analysis more operational and reliable, correlates should be further divided into high-and low-evidence correlates. The high-evidence correlates are defined as correlates of high investigation frequency (beyond 10 effect sizes), and the low-evidence correlates are of low investigation frequency (5–9 effect sizes). Third, it is premature to execute moderator analysis for the low-evidence correlates thus far, potential moderators are identified from the literature and moderator analysis should only be done for the high-evidence correlates (Li, 2022a). In this study, we have identified two high-evidence correlates (foreign language writing performance and foreign language writing self-efficacy) and three low-evidence correlates (foreign language listening anxiety, foreign language speaking anxiety and foreign language reading anxiety) of foreign language writing anxiety. The main correlates and potential moderators of the high-evidence correlates are defined in the remainder of this section.

#### High-evidence correlates and moderators

##### Foreign language writing performance and correlates

As a frequently examined correlate, foreign language writing performance (*writing performance* hereafter) refers to those studies that reported foreign language learners’ writing scores or grades. For instance, Liu and Ni (2015) investigated the foreign language writing anxiety–writing performance correlation among 1,174 first-year Chinese university EFL learners of intermediate level, and obtained a weak and negative correlation (*r* = [−0.136, −0.091], *p* < 0.001), corroborating the interview result that “around one third of the learners did not report having anxiety when writing in a foreign language” (p. 55), suggesting that writing anxiety might not be so influential to learners’ writing performance. Zhang (2011) examined the foreign language writing anxiety–writing performance correlation among Chinese English majors of high proficiency level, and reported a moderately negative effect (*r* = [−0.879, −0.838], *p* < 0.001). The non-consensual results may be explained by such potential moderators as language proficiency, target language, age and types of anxiety (e.g., Zhang, 2019; Li, 2022a). Consequently, this study first calculates the aggregated foreign language writing anxiety–writing performance correlation, and then reports the moderator results of language proficiency, target language, age and types of anxiety.

##### Foreign language writing self-efficacy and correlates

The operational definition of foreign language writing self-efficacy (*writing self-efficacy* hereafter) refers to learners’ self-confidence in the ability to succeed in foreign language writing (Cheng, 2004). Since Cheng (2004), the foreign language writing anxiety–writing self-efficacy correlation has caught the attention of many researchers (Li and Liu, 2013; Tola and Sree, 2016; Guo, 2018). These studies regarding the significant foreign language writing anxiety–writing self-efficacy correlation have been confirmed in some (e.g., *r* = [−0.760, −0.382], *p* < 0.001, Li et al., 2013; and *r* = [−0.420, −0.360], *p* < 0.001, Cheng, 2004), but not in others (e.g., *r* = 0.186, *p* > 0.050, Singh and Rajalingam, 2012), which may give rise to potential moderators, including language distance, target language, language proficiency and types of anxiety (e.g., Teimouri et al., 2019; Botes et al., 2020; Li, 2022a).

#### Low-evidence correlates

##### Foreign language listening anxiety

Foreign language listening anxiety (*listening anxiety* hereafter) refers to the “fear of misunderstanding what language learners listen to and being embarrassed by interpreting the message wrongly” (Serraj and Noordin, 2013, p. 3). Language learners who are anxious about their listening comprehension might experience the lack of confidence and worry over foreign language listening tasks, or even “failure to recognize spoken foreign language words” (Bekleyen, 2009, p. 664). The significantly positive foreign language writing anxiety–listening anxiety correlation suggests that foreign language learners with high writing anxiety are likely to feel higher listening anxiety, and the vice versa (Xiao and Wong, 2014; Cheng, 2017), justifying the needs to have a fresh look at the role of listening anxiety by exploring the foreign language writing anxiety–listening anxiety correlation.

##### Foreign language speaking anxiety

Foreign language speaking anxiety (*speaking anxiety* hereafter) is defined as a sense of fear or anxiety that language learners would feel when using, speaking or communicating in a foreign language (Woodrow, 2006). The foreign language writing anxiety–speaking anxiety correlation has been gaining attention among researchers (e.g., Gkonou, 2011; Xiao and Wong, 2014; Cheng, 2017). For instance, Cheng (2017) recruited 523 Chinese college students to measure the correlations among four foreign language-skill-specific anxieties, and found the foreign language writing anxiety–speaking anxiety correlation was *r* = 0.510, *p* < 0.050. In another study, Gkonou (2011) also surveyed the correlation and found *r* = [0.340, 0.543], *p* < 0.050, While these primary studies sheds light on the important role of speaking anxiety, little is known about the average correlation, necessitating a meta-analysis to aggregate the effects with larger sample sizes.

##### Foreign language reading anxiety

Foreign language reading anxiety (*reading anxiety* hereafter) is defined as the “perceptions of uneasiness, apprehension or stress from which an individual might suffer when reading a foreign language text” (Capan and Karaca, 2013, p. 1362). Researchers investigated the foreign language writing anxiety–reading anxiety correlation, and found that the correlation was moderate and positive: *r* = [0.272, 0.546], *p* < 0.050 (Xiao and Wong, 2014) and *r* = 0.580, *p* < 0.050 (Cheng, 2017). The moderate foreign language writing anxiety–reading anxiety correlation in these primary studies calls for more investigations. For this reason, we take reading anxiety as the correlate of writing anxiety.

### Research statements and questions

The current study aims to achieve two research purposes. First, we carried out a meta-analysis based on a systematic review of existing primary studies that explored the correlations of foreign language writing anxiety and its two high-evidence correlates (foreign language writing self-efficacy and foreign language writing performance) along with three low-evidence correlates (foreign language listening anxiety, foreign language speaking anxiety and foreign language reading anxiety). Second, apart from the correlations under investigation, we also examined the moderating effects of learners’ age, language proficiency, target language, types of anxiety and language distance for the high-evidence correlates. To this end, the following research questions are to be addressed.

***Research question 1***: What are the correlations of foreign language writing anxiety and two high-evidence correlates (writing performance and writing self-efficacy)?

***Research question 2***: How do age, language proficiency, target language, types of anxiety and language distance moderate the correlations of writing anxiety and its two high-evidence correlates (writing performance and writing self-efficacy)?

***Research question 3***: What are the correlations of foreign language writing anxiety and three low-evidence correlates (listening anxiety, speaking anxiety and reading anxiety)?

## Research method

### Literature search and inclusion criteria

The study attempts to retrieve the currently available literature of writing anxiety in second and/or foreign language learning published during 2000 to 2021, because foreign language writing research remained few in number before 2000 (Cheng, 2002, 2004). Several electronic databases (e.g., Chinese CNKI, ERIC, ProQuest, ScienceDirect, Springer, web of science, Wiley) and search engines (Chinese Baidu Scholar and Google Scholar) were retrieved with a combination of the following key words: *affect*, *foreign language*, *second language (L2)*, *self-efficacy*, *(writing) score*, *(writing) grade*, *(writing) achievement*, *(writing) proficiency*, *listening anxiety*, *speaking anxiety*, *reading anxiety*, *writing anxiety* and *writing apprehension*. Moreover, to ensure the comprehensiveness of the literature, we conducted backward and forward citation searches based on seminal article (Daly and Miller, 1975; Cheng, 2004) and “snowballing technique” (Biernacki and Waldorf, 1981) by scanning references in the identified articles. To ensure the quality of primary literature during the selection process, only the peer reviewed journal articles, dissertations, and conference proceedings were included. The inclusion/exclusion criteria were proposed as follows:

1. The study should investigate the correlations of second or foreign language writing anxiety, writing performance (writing test, score or grade), writing affects and other skill-specific anxieties, resulting in 30 primary studies included.
2. The study should contain the statistics (e.g., correlation, sample sizes, standard error and variance, etc.) sufficient for the transformation or calculation of effect sizes. Eighteen articles were included by excluding 12 publications that failed to provide the sufficient statistics for calculation.
3. The backward and forward together with snow-balling searches from the existing studies (e.g., Daly and Miller, 1975; Cheng, 2004; Woodrow, 2011) on the section of literature review, together with specific search of each correlates yielded another three journal articles and one conference proceeding on foreign language writing anxiety needed for the forthcoming analysis.
4. Both peer-reviewed journal articles or unpublished materials (e.g., conference proceedings, master’s or doctoral dissertations) were retrieved, which resulted in 22 primary studies.

### Variables coded for each study

According to Wilson (2019, p. 154), a coding scheme should “capture the pertinent information suitable for meta-analysis.” Thus, the selected studies were coded in terms of related correlates (writing performance, writing self-efficacy, listening anxiety, speaking anxiety and reading anxiety) and moderators (types of anxiety, age, target language, language distance, and language proficiency). The code scheme proposed was presented in Table 2, including the following major categories:

**Table 2:** Coding scheme.

Coding types	Subtypes	Operational definitions	References
Correlates			
Foreign language writing performance	Writing grade/score	Studies that reported learners’ writing score or grade	Brown et al. (2018); Cheng (2004)
Foreign language writing self-efficacy	Self-efficacy in writing	Students’ self-confidence in the ability to succeed in foreign language writing	Cheng (2004)
Foreign language listening anxiety	Listening anxiety	Studies that reported listening anxiety as a correlate of writing anxiety.	Kim (2000)
Foreign language speaking anxiety	Speaking anxiety	Studies that reported speaking anxiety as a correlate of writing anxiety	Woodrow (2006)
Foreign language reading anxiety	Reading anxiety	Studies that reported reading anxiety as a correlate of writing anxiety	Saito et al. (1999)
Moderators			
Types of anxiety	Overall anxiety	Studies that reported anxiety in general	Cheng (2004)
	Avoidance behavior	Students’ avoidance of writing in a foreign language	
	Cognitive anxiety	Students’ perceptual arousal to write in a foreign language	
	Somatic anxiety	Students’ physiological arousal to write in a foreign language.	
Age	Child/Adolescent	Less than grade twelve (age 18)	Researcher-designed
	Adult	At and over grade twelve (18 or older)	
Target language	English	English as a foreign language (EFL)	Levine (2003)
	Mixed languages	Other mixed languages	
Language distance	Near	Indo-European L1 and Indo-European L2	Lervåg and Lervåg (2011)
	Distant	Indo-European L1 and non-Indo-European L2 or Non-Indo-European L1 and Indo-European L2	
Language proficiency	High	Studies that reported high (highly proficient learners), intermediate (intermediate learners) and low (foreign language beginners) level	Li (2022a)
	Intermediate		
	Low		

Coding procedures were followed to ensure the methodological quality (e.g., Valentine, 2019): On the one hand, as issue of data dependencies should be considered first (Plonsky and Oswald, 2014), multiple studies reported in a single paper involving different types of measurement or participants were coded separately as independent studies. On the other hand, to ensure the reliability of coding scheme, two coders who had a consistent understanding of coding types, subtypes and operational definitions were required to independently code the items. They should also negotiate with each other when discrepancies occurred.

### Calculation and analysis of the effect sizes

For data calculation and interpretations, correlation coefficients, sample sizes and effect directionality were first converted to Fisher’s z, and the aggregated coefficients, standard error and confidence interval were then calculated. According to Plonsky and Oswald (2014), the interpretations of the effect size were indexed as 0.25 (small), 0.40 (moderate), and 0.60 (large), respectively.

For the data analysis, both fixed and random model were utilized to compute all the aggregated correlations, depending on the different sources of variation in effect sizes. For the fixed model, all studies were assumed to share a common true effect and the between-study variation of the effect sizes is sampling error. By contrast, for the random model, the true effects were assumed to have been sampled from a between-study variation across studies (Borenstein et al., 2009; Plonsky and Oswald, 2012). As such, a random model was consulted, and the heterogeneity was located in respect to moderators including age, language proficiency, target language, types of anxiety and language distance.

## Results

Results were reported based on the 84 effect sizes with a total of 24,290 participants involved (*M* ± *SD* = 289.167 ± 333.380, range = 50–1,635). In the rest of this section, results of high-evidence correlates and moderator analysis were first reported, and then results of three low-evidence correlates followed suit. As number of low-evidence correlates was too small to analyze the moderating effects, moderator analysis for the low-evidence correlates was not executed accordingly (*cf.* Lervåg and Lervåg, 2011).

### Results of high-evidence correlates and moderator analysis

#### Foreign language writing anxiety and writing performance

Forty effect sizes consisting of 14,918 participants (*M* ± *SD* = 372.950 ± 436.800, range = 50–1,635) examined the foreign language writing anxiety–writing performance correlation.

As presented in Table 3, the foreign language writing anxiety–writing performance correlation was significantly moderate, *r* = −0.298, 95% CI [−0.353, −0.240], *z*(39) = −9.667, *p* < 0.001. No any publication bias was observed, *N_fs_* = 7,503 > *N_observed_* = 40, *p* < 0.001, which did not affect the results.

**Table 3:** Overall average correlation and publication bias test for the high-evidence correlates.

Correlates	*r* [95% CI]	*N_fs_*	*N_observed_*	*r_adjusted_*
Writing performance	−0.298 [−0.353, −0.240]	7,503	40	−0.185 [−0.200, −0.169]
Writing self-efficacy	−0.382 [−0.456, −0.302]	3,715	19	−0.348 [−0.371, −0.324]

*N_fs_* = number of missing studies that would bring *p* > 0.05; *N_observed_* = number of observed studies.

As both significance tests [*Q*(39) = 520.779, *p* < 0.001, *I^2^* = 92.511] were significantly heterogenous, several moderator analyses should be further conducted. Results of the moderator analysis regarding language proficiency, target language, age and types of anxiety were reported in the rest of this section, respectively.

##### Language proficiency

As shown in Table 4, language proficiency significantly moderates the foreign language writing anxiety–writing performance correlation (*Q*_between_ = 11.408, *p* = 0.003). Pairwise comparisons showed that, the low proficiency learners had the weakest foreign language writing anxiety–writing performance correlation (*r* = −0.129, 95% CI [−0.219, −0.037]), which was statistically lower than high proficiency learners (*Q*_between_ = 7.553, *p* = 0.006), and intermediate proficiency learners (*Q*_between_ = 8.939, *p* = 0.003). No significant difference was found between high proficiency learners and intermediate proficiency learners (*Q*_between_ = 0.225, *p* = 0.635).

**Table 4:** Moderator analyses for the foreign language writing anxiety–writing performance correlation.

Moderators	*k*	*r* [95% CI]	*I^2^*	Heterogeneity		
				*Q*	*df*	*p*
*Language proficiency*	40	−0.252 [−0.302, −0.201]	92.511	11.408**	2	0.003
High	10	−0.337 [−0.444, −0.219]	83.799			
Intermediate	27	−0.304 [−0.373, −0.232]	94.245			
Low	3	−0.129 [−0.219, −0.037]	48.383			
*Target language*	19	−0.366 [−0.432, −0.295]	91.137	0.731	1	0.393
English	12	−0.409 [−0.521, −0.284]	93.341			
Mixed languages	7	−0.345 [−0.427, −0.259]	82.748			
*Age*	40	−0.312 [−0.362, −0.259]	92.511	3.793△	1	0.051
Adult	34	−0.280 [−0.340, −0.218]	93.113			
Child/Adolescent	6	−0.393 [−0.481, −0.297]	49.695			
*Types of anxiety*	27	−0.249 [−0.337, −0.171]	92.511	1.134	2	0.567
avoidance behavior	13	−0.256 [−0.337, −0.171]	80.303			
cognitive anxiety	7	−0.222 [−0.298, −0.143]	66.879			
somatic anxiety	7	−0.188 [−0.280, −0.092]	76.654			

△*p* < 0.100; *p < 0.050; **p < 0.010. Unreported information is not included.

##### Target language

Target language involves two types, *viz.* English and mixed languages. It could be found in Table 4, target language obtained no statistically significant moderating effect on the foreign language writing anxiety–writing performance correlation (*Q*_between_ = 0.225, *p* = 0.635).

##### Age

Age was found to significantly moderate the foreign language writing anxiety–writing performance correlation (*Q*_between_ = 3.793, *p* = 0.051), indicating that children and adolescents (*r* = −0.393, 95% CI [−0.481, −0.297]) tend to have more negative foreign language writing anxiety–writing performance correlation than adults (*r* = −0.280, 95% CI [−0.340, −0.218]).

##### Types of anxiety

According to Cheng (2004), foreign language writing anxiety could be further classified into three types, i.e., avoidance behavior, cognitive anxiety and somatic anxiety. As apparent in Table 4, there is no significant moderating effect of types of anxiety (*Q*_between_ = 1.134, *p* = 0.567).

#### Foreign language writing anxiety and writing self-efficacy

Nineteen effect sizes involving 5,626 participants (*M* ± *SD* = 296.105 ± 152.918, range = 50–738) explored the foreign language writing anxiety–writing self-efficacy correlation.

As apparent in Table 3, the foreign language writing anxiety–writing self-efficacy correlation was moderate, *r* = −0.382, 95% CI [−0.456, −0.302], *z*(18) = −8.717, *p* < 0.001. No publication bias could be observed, *N_fs_* = 3,715 > *N_observed_* = 19, *p* < 0.001, which did not affect the results.

As both significance tests [*Q*(18) = 203.084, *p* < 0.001, *I^2^* = 91.137] were reported, necessitating further moderator analyses. Moderator analyses regarding language distance, target language, language proficiency and types of anxiety were reported in Table 5, which indicated that no moderator surveyed above could reliably explain the variation of foreign language writing anxiety–writing self-efficacy correlation.

**Table 5:** Moderator analyses for the foreign language writing anxiety–writing self-efficacy correlation.

Moderators	*k*	*r* [95% CI]	*I^2^*	Heterogeneity		
				*Q*	*df*	*p*
*Language distance*	19	−0.378 [−0.431, −0.322]	91.137	0.018	1	0.893
Distant	16	−0.377 [−0.430, −0.322]	79.254			
Similar	3	−0.415 [−0.795, 0.201]	98.371			
*Target language*	19	−0.366 [−0.432, −0.295]	91.137	0.731	1	0.393
English	12	−0.409 [−0.521, −0.284]	93.341			
Mixed languages	7	−0.345 [−0.427, −0.259]	82.748			
*FL proficiency*	19	−0.367 [−0.444, −0.284]	91.137	0.898	1	0.343
High	2	−0.588 [−0.857, −0.068]	94.276			
Intermediate	17	−0.361 [−0.439, −0.277]	91.164			
*Types of anxiety*	8	−0.400 [−0.439, −0.360]	68.527	3.068	2	0.216
avoidance behavior	4	−0.490 [−0.599, −0.363]	83.504			
cognitive anxiety	2	−0.411 [−0.467, −0.350]	0.000			
somatic anxiety	2	−0.370 [−0.429, −0.308]	0.000			

Unreported information is not included.

### Results of low-evidence correlates

Results of three low-evidence correlates of foreign language writing anxiety (listening anxiety, speaking anxiety and reading anxiety) were reported in Table 1, but moderator analyses were performed as there was no sufficient data for aggregation (Li, 2022a).

Five effect sizes comprising 871 participants dealt with the foreign language writing anxiety–listening anxiety correlation. As shown in Table 1, the correlation result was significantly moderate and positive, *r* = 0.481, 95% CI [0.430, 0.528], *z*(4) = 11.849, *p* < 0.001.

Nine effect sizes comprising a total of 1,383 participants investigated the foreign language writing anxiety–speaking anxiety correlation. As shown in Table 1, the correlation was significantly moderate and positive, *r* = 0.455, 95% CI [0.377, 0.526], *z*(8) = 10.213, *p* < 0.001.

Likewise, five effect sizes consisting of 871 participants explored the foreign language writing anxiety–reading anxiety correlation. As shown in Table 1, the correlation was significantly moderate and positive, *r* = 0.489, 95% CI [0.376, 0.588], *z*(4) = 7.506, *p* < 0.001.

## Discussion

The present study endeavored to quantitatively meta-analyze the two high-evidence correlates (writing performance and writing self-efficacy) and the three low-evidence correlates (listening anxiety, speaking anxiety and reading anxiety) of foreign language writing anxiety identified in the primary literature. Simultaneously, it also dealt with moderator analyses for the two high-evidence correlates, including learners’ age, foreign language proficiency, target language and language distance.

Research question 1 explored the correlations of foreign language writing anxiety and its two high-evidence correlates (writing performance and writing self-efficacy). As noted, the results demonstrated that foreign language writing anxiety has a moderate correlation with writing performance and writing self-efficacy, suggesting that worse writing performance is likely to be accompanied with higher writing anxiety, and those with higher writing anxiety tend to have a lower writing self-efficacy, mirroring an increasing number of studies maintaining the detrimental or debilitative effects of foreign language writing anxiety (e.g., Horwitz, 2017; MacIntyre, 2017). A plausible explanation might be that, those learners with higher writing anxiety might lead to the lack of self-confidence and ability to retrieve linguistic (i.e., lexical, semantic and syntactic) knowledge from the mental lexicon, choose appropriate language structure, use appropriate rhetorical devices and adopt other writing skills, then perfectly organize and output ideas as required, which would also result in unsatisfactory writing performance and a low sense of self-efficacy in writing tasks in turn (Öztürk and Saydam, 2014; Kırmızı and Kırmızı, 2015; Tola and Sree, 2016).

Research question 2 concerned the moderating effects of age, language proficiency, target language, types of anxiety and language distance on the correlations of foreign language writing anxiety and two high-evidence correlates. The results of moderator analysis suggested that learners’ age and language proficiency, rather than target language, types of anxiety and language distance, are found to be significant moderators. Specifically, compared with the two higher proficiency learners, those low proficiency learners had the weakest foreign language writing anxiety–writing performance correlation, and no difference was found between the intermediate and high proficiency learners, resonating the argument that writing anxiety may change as a function of language proficiency (Zhang, 2019). In other words, compared with relatively high proficiency peers (*viz*. intermediate and high proficiency learners), low proficiency learners might not perform competently in writing tasks that invoke high loads of working memory and are unlikely to be actively involved in foreign language writing, hence their writing anxiety might not be triggered. A further support could be found in Horwitz (1996) who asserted that even the highly proficient language learners may experience anxiety when using a foreign language. The finding, however, is inconsistent with that of Zhang (2019) who did not find the moderating effects of language proficiency on foreign language anxiety–language performance correlation. A possible explanation for the inconsistence may rest on the difference in anxiety: domain-general language anxiety vs. skill-specific writing anxiety. In other words, while Zhang (2019) deals with the relationship between domain-general language anxiety and language proficiency, writing tasks in this meta-analysis that involves the skill-specific writing anxiety would be more cognitive resources demanding compared to language-general tasks or other receptive tasks in terms of different degree of task difficulty (He, 2018), adding to the emerging body of literature by showing the significant moderating effect of language proficiency.

Meanwhile, our meta-analysis provides another piece of evidence that the writing anxiety–writing performance correlation is sensitive to age effect, as reflected in the results that children and adolescents tended to have more negative correlations between foreign language writing anxiety and writing performance than adults. This finding, however, is not in line with Zhang (2019, p. 12) who claims “the language anxiety–language performance correlation became stronger as age increased.” The discrepancy might reside in the different measures of anxiety, since Zhang’s (2019) meta-analysis involves language anxiety–language performance correlation, while our study deals with writing anxiety–writing performance correlation. Another explanation for the significant age effect is that, adults’ cognitive or metacognitive skills tend to be more mature to reduce foreign language writing anxiety compared to children and adolescents (Li, 2022a).

Some nonsignificant moderating effects of target language, types of anxiety and language distance should be noteworthy as well. First, regarding target language (English vs. mixed languages), no moderating effect was found on the writing anxiety–writing performance correlation, indicating that learners whose writing anxiety–writing performance correlation might not vary across different target languages. This result could be explained by the complex and demanding nature of the writing process, that is, it is not the target language, be it English or other languages, that matters, rather a complex writing process that matters (Kim and Kim, 2020). Second, the moderating effect regarding types of anxiety was not found on both the writing anxiety–writing performance correlation together with the writing anxiety–writing self-efficacy correlation, suggesting that somatic anxiety, cognitive anxiety and avoidance behavior might play a somewhat equal role in the aforementioned correlations. Third, inspired by Lervåg and Lervåg (2011) who meta-analyzed reading comprehension and its correlates, this study also examined the moderating effect of language distance regarding the orthographic difference/similarity between first and foreign language. Contrary to Lervåg and Lervåg (2011), our study obtained no significant moderating effect of language distance. A plausible explanation for such a discrepancy might lie in the difference between reading and writing modal. As for reading modal, the materials would be visually presented first. In this case, the orthographic difference/similarity of the visually presented materials might play a moderating role, whereas writing modal involves a series of complex activities, e.g., how to generate, organize ideas and how to organize the ideas in written forms. As such, the moderating effect regarding the distance between first and foreign language visually presented might not be the same case as in reading comprehension (Lervåg and Lervåg, 2011).

Research question 3 dealt with the correlations between foreign language writing anxiety and three low-evidence correlates (listening anxiety, speaking anxiety and reading anxiety). The moderate and positive correlations of the three low-evidence correlates have been obtained, resonating the previous studies that investigated different types of language learners, e.g., Korean EFL learners (Pae, 2013), Chinese-as-a-heritage-language (CHL) learners (Xiao and Wong, 2014) and Korean-as-a-heritage-language (KHL) learners (Jee, 2016), confirming the “interdependence among the four skill-specific anxieties” (Pae, 2013, p. 250).

Taken together, the pedagogical implications both for researchers and teachers in the field to help alleviate learners’ writing anxiety are also inferred as follows. First, as the debilitative effects of foreign language writing anxiety have been found with regard to writing performance and writing self-efficacy, teachers should try to locate the sources of learners’ writing anxiety. One implication is that teachers could establish a relaxed learning environment, design relaxation writing activities and encourage students to express their fears (Li, 2022a). Another way is to seek for some automated writing evaluation (AWE) tools that enable to provide foreign language learners with timely and supportive writing feedback (Li et al., 2019). By introducing online pedagogical intervention along with face-to-face instruction (Li, 2022c), those shy learners might be likely to feel less anxious to express their anxieties and personalized needs. Second, as the moderating effects of learners’ individuality (e.g., age and language proficiency) were found to be significant, when teaching how to write well, teachers should try to alleviate learners’ writing anxiety with a particular eye on learners’ personalized needs. For instance, teachers normally offer special guidance to those underachievers. Our finding suggests their attention should also be paid equally to the intermediate and advanced proficiency learners, since they might also experience tremendous anxiety when writing in a foreign language (Horwitz, 1996). Third, the interdependence among the four skill-specific anxieties suggests that foreign language researchers and practitioners should pay equally balanced attention to anxieties arising from each of the four language skills (Pae, 2013).

## Conclusion

Motivated by the earlier attempts, this study aims to understand the correlates of foreign language writing anxiety. Results showed that foreign language writing anxiety has moderate correlations with writing performance and writing self-efficacy. Besides, as compared target language, types of anxiety and language distance, significant moderating effects of learners’ age and language proficiency have been obtained. The three low-evidence correlates have moderate effect sizes, with speaking anxiety, reading anxiety and listening anxiety being the moderate and positive correlate.

One potential limitation should be addressed though. Considering the needs of sufficient information from the primary studies, the current study only included limited correlates (writing performance, writing self-efficacy, listening anxiety, speaking anxiety, and reading anxiety) and moderators (learners’ age, language proficiency, target language, types of anxiety and language distance). To gain a fuller understanding, future study should consider other equally important correlates (e.g., motivation, strategy and attitude) and moderators (e.g., gender and learning style) of foreign language writing anxiety.

## Data availability statement

The data analyzed in this study is subject to the following licenses/restrictions: Data will be publically available upon reasonable request. Requests to access these datasets should be directed to RL, liruidianzi@hotmail.com.

## Author contributions

The author confirms being the sole contributor of this work and has approved it for publication.

## Funding

This study was funded by the Social Science Foundation of Hunan Province (grant number 20ZDB005).

## Conflict of interest

The author declares that the research was conducted in the absence of any commercial or financial relationships that could be construed as a potential conflict of interest.

## Publisher’s note

All claims expressed in this article are solely those of the authors and do not necessarily represent those of their affiliated organizations, or those of the publisher, the editors and the reviewers. Any product that may be evaluated in this article, or claim that may be made by its manufacturer, is not guaranteed or endorsed by the publisher.
